# A prospective observational cohort study of posterior tibial nerve stimulation in patients with multiple sclerosis: design and methods

**DOI:** 10.1186/s12894-020-00629-y

**Published:** 2020-05-27

**Authors:** Giulia I. Lane, Yang Mao-Draayer, Paholo Barboglio-Romo, J. Quentin Clemens, Priyanka Gupta, Rod Dunn, Yongmei Qin, Anne P. Cameron, John T. Stoffel

**Affiliations:** 1 Department of Urology, University of Michigan, 1500 East Medical Drive, Taubman Center 3875, Ann Arbor, MI 48109 USA; 2 Department of Neurology, University of Michigan, Ann Arbor, MI USA

**Keywords:** Quality of life, Urinary incontinence, Neuromodulation, Multiple sclerosis, Prospective studies, Tibial nerve, Urinary bladder, Neurogenic

## Abstract

**Background:**

Posterior tibial nerve stimulation (PTNS) is a promising treatment for lower urinary tract symptoms (LUTS) in patients with MS. However, long term data focusing on PTNS impact on health-related quality of life (HRQOL), bowel and bladder symptoms are lacking. This paper describes a study protocol that examines the extended efficacy of PTNS on MS related bladder and bowel symptoms and resulting HRQOL.

**Methods/Design:**

This is a single-centered, prospective, longitudinal, observational cohort study of patients with MS who suffer from LUTS and are refractory to two prior treatment modalities. Participants who have elected to pursue PTNS therapy for LUTS will be eligible. The primary outcome is the median number of urinary frequency and incontinence episodes on a 3-day voiding diary at 3, 12 and 24 months compared to baseline. Secondary outcome measures will include change in total AUA-SS, M-ISI, NBSS, SF-12, SSS and BCS scores from baseline The Expanded Disability Status Scale and magnetic resonance imaging will be evaluated at baseline and annually throughout the study.

**Discussion:**

This research protocol aims to expand on the existing literature regarding outcomes of PTNS in MS. Specifically, it will provide long term follow-up data on bladder, bowel, sexual and HRQOL outcomes. The completion of this study will provide longitudinal efficacy data of the impact of PTNS in MS patients.

**Trial registration:**

NCT04063852.

## Background

Multiple Sclerosis (MS) is the most common autoimmune neuro-inflammatory disease of the central nervous system and affects approximately 31 per 100,000 Americans annually, with 700,000 Americans living with MS in 2010 [1].

MS is characterized by four primary disease courses: clinically isolated syndrome (CIS), relapsing remitting (RRMS), secondary progressive (SPMS) and primary progressive (PPMS) [2, 3]. Clinically isolated syndrome is a pre-MS syndrome and is described as the initial episode of neurologic deficit which may progress to MS. [2] In some patients, CIS precedes the diagnosis of relapsing-remitting MS (RR-MS). Relapsing-remitting MS (RRMS) is characterized by discrete episodes of neurological dysfunction or relapses, with no interim disease progression. Patients may fully recover from a relapse or may experience residual deficits. Secondary progressive MS patients have gradual neurological deterioration (progression) with increasing physical debilitation following a period of relapsing remitting disease [4]. Finally, primary-progressive MS (PPMS) is defined as steady disease progression from its onset and may include periods of stable disease or temporary symptomatic improvement [5].

Lower urinary symptoms (LUTS) including overactive bladder (OAB) are reported in 75–90% of all MS patients, and can be very bothersome to patients [6]. However, stage and type of disease may impact perception and severity of urinary symptoms in MS patients. Utilizing urodynamic data, others showed that there is high prevalence of urinary symptoms even in clinically isolated syndrome (CIS), before greater neurologic impact from the disease is found [7]. Once patients have established disease, urinary symptoms may be perceived differently based on rapidity of disease progress.

**Fig. 1:**
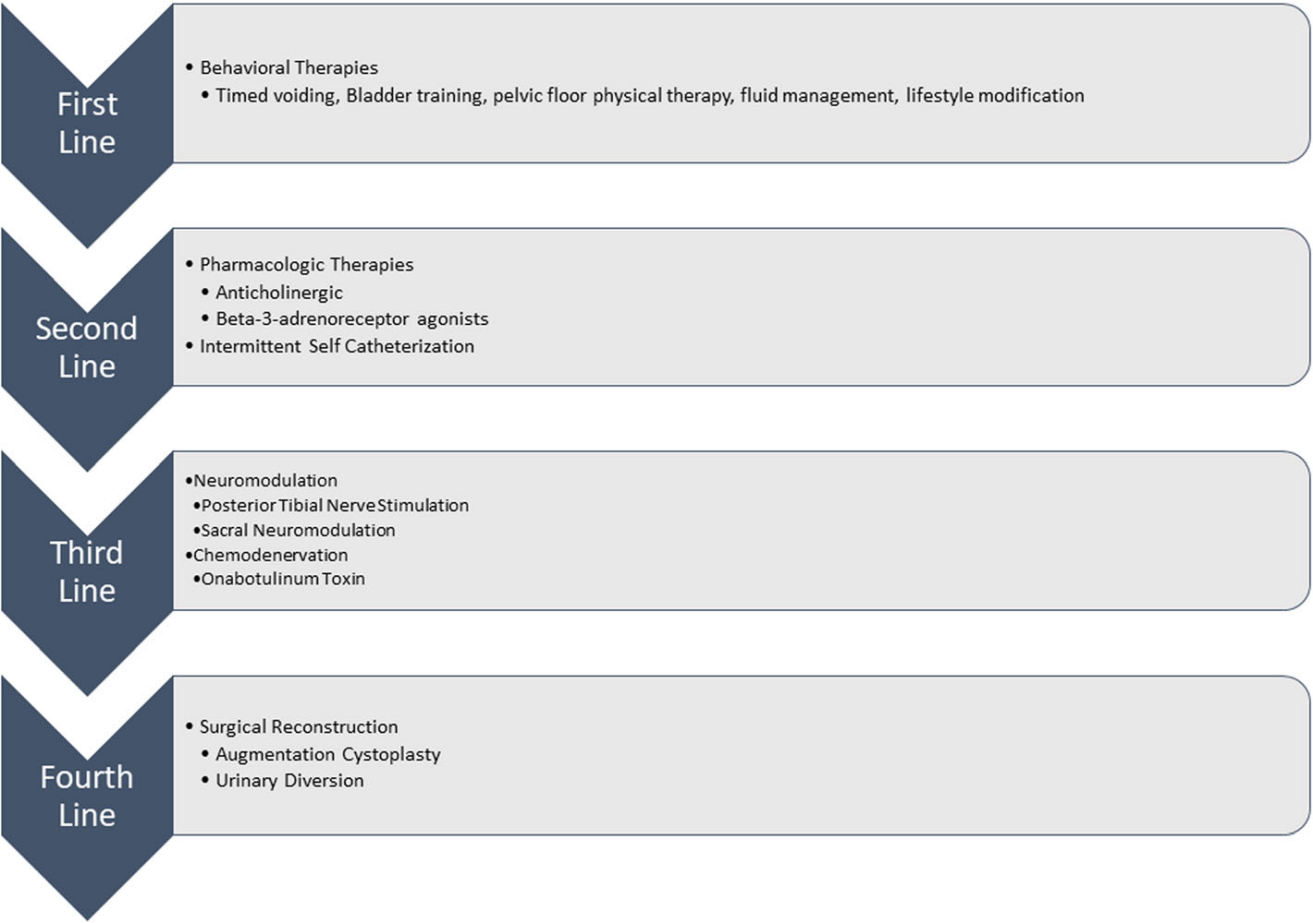
Management of Lower Urinary Tract Symptoms in Patients with Multiple Sclerosis

Current management of MS related LUTS follows the overactive bladder pathway: first line lifestyle modifications, second line adjunctive pharmacotherapy, third line neuromodulation (sacral or posterior tibial) or chemodenervation of the bladder (onabotulinum toxin) and fourth line invasive surgical reconstruction [8–10]. The implementation of intermittent or indwelling catheter can be an adjunct to these therapies (Fig. 1).

Each intervention can have significant quality of life implications. For example, second line pharmacotherapy with anticholinergic is often poorly tolerated because it can exacerbate symptoms of dry mouth, constipation and central nervous system side effects like confusion [8]. Likewise, the addition of intermittent catheterization can add complexity to the patient’s lifestyle with unclear impact on decreasing LUTS [11]. In regards to third line therapy, intradetrusor onabotulinumtoxin (Botox) can be effective at controlling symptoms, but it requires cystoscopy to administer, the treatment needs to be repeated every 6–12 months, and each administration carries a risk of urinary tract infection and urinary retention [12].

Neuromodulation, another third line therapy, includes both sacral neuromodulation and posterior tibial nerve stimulation. Sacral neuromodulation involves 1 or 2 surgeries for placement of a neuromodulation lead and an implantable pulse generator to stimulate the S3 nerve root. Limited data suggest sacral neuromodulation may be effective for MS related LUTS, however the sacral neuromodulation leads are not MRI compatible [8, 13, 14]. Since many patients with MS require frequent MRI for surveillance, this poses a barrier for use of current implantable sacral neuromodulation technology. In these patients, percutaneous posterior tibial nerve stimulation (PTNS) offers a minimally invasive alternative to relieve bladder symptoms via similar pathways as sacral neuromodulation.

Posterior tibial nerve stimulation, involves placement of a solid stainless-steel needle into the posterior tibial nerve and uses this to conduct a fixed electrical pulse through the nerve. The posterior tibial nerve derives from the L4-S3 nerve roots, neuromodulation of this nerve has been shown to improve overactive bladder symptoms [15]. The office-based therapy is performed weekly for 12 weeks during 30-min sessions; patients who have improvement in symptoms can proceed to maintenance treatments at regularly scheduled intervals.

Only seven clinical studies have been published regarding the use of PTNS in patients with MS and refractory LUTS [16–22]. (Table 1) Of these studies, four represent prospective case series evaluating the efficacy of PTNS for LUTS in MS, with a total of 131 patients [18–20, 22]. These studies reported significant improvement in LUTS with overall the rate of improvement (defined as greater than 50% improvement in symptoms) between 33 and 89% (Table 1).

**Table 1:** Literature review of Posterior Tibial Nerve Stimulation in Multiple Sclerosis

Author	Year	N	Inclusion Criteria*	Follow-up (months)	Primary Outcome Results
Fjorback [1]	‘07	8	UDS: DO and MCC < 300 mL	n/a	• PTNS unable to suppress detrusor contraction. • No reduction of urgency • No difference in peak detrusor pressure • Median volume at first contraction was 36% higher with stimulation. (*p* = 0.0078)
Kabay [2]	‘08	29	storage symptoms	n/a	• Mean volume first involuntary contraction increased from 183 to 230 mL. (*p* < 0.001) • Mean maximum cystometric capacity increased from 193 to 286 mL. (*p* < 0.001)
Kabay [3]	‘09	19	LUTS	3	• Complete Response rates > 50% improvement: urgency (33%), incontinence (40%), frequency (58%), nocturia (75%), pad test (90%) of patients • Partial Response between 25 and 50% improvement:urgency (53%), incontinence (50%), frequency (26%), nocturia (25%), pad test (10%) of patients • UDS response: DSD resolved in 3/5, significant improvement after PTNS in first involuntary detrusor contraction volume, MCC, Qmax, PVR (*p* < 0.05)
de Seze [4]**	‘11	70	refractory OAB symptoms	3	• 30d: 51% resolution of severe urgency, 67% reduction in frequency by 3 episodes/day • 90d: improvement in 83.3% of patients in 3 day bladder diary, MHU or warning time
Gobbi [5]	‘11	18	refractory LUTS	3	• Improved frequency, nocturia, Increased voided volume, • PPBC decreased by 3 points (*p* = 0.003), PPIUS decreased by 2 points (*p* = 0.005), UB-VAS decreased by 4 cm (p = 0.005), improvement in KHQ (p < 0.05)
Zecca [6]	‘14	initial: 83 Maint: 74	refractory LUTS, UDS with DO, UAB or DSD	24	• 60% (44/74) required maintenance every 2 weeks, 4% required maintenance every week, mean treatment was: 1.79 sessions/patient/month • 82% of patients reported treatment satisfaction on TS-VAS, GRA, and PPBC at 24 months
Zecca [7]	‘14	83	refractory LUTS, UDS with Dom UAB or DSD	3	• Sensory response: 64%, Motor response: 6%, Sensory-Motor: 30%; Sensory response with or without motor response was associated with better outcome than motor alone (p < 0.001)
Canbaz- Kabay [23]	‘17	initial: 29 Maint: 21	refractory OAB	12	• Improvement in frequency, nocturia, urgency (all p < 0.001) and voided volume improved mean 72 cc (p < 0.05). • Change from baseline on the ICIQ-SF, OABv8 and OAB-q: decreased symptom severity and health related quality of life (*P* < 0.001)

*all studies included adults with multiple sclerosis

**Study of Transcutaneous PTNS versus others, which were percutaneous

*UDS* urodynamics, *DO* detrusor overactivity, *MCC* Maximum Cystometric Capacity, *LUTS* Lower urinary tract symptoms, *Maint* Maintenance, *NR* Not reported

Despite these findings, the studies were limited by their short term follow up (range 3–12 months), strict inclusion criteria, and lack of important quality of life outcomes. Outcomes beyond 12 months and the impact of standard maintenance therapy have only been studied in one prior study [20]. Moreover, there is limited research on the effects of PTNS on related pelvic organ dysfunction such as bowel and sexual function among patients with MS. [23, 24] As PTNS requires significant investment on the part of patients and clinicians, further studies generating more robust evidence regarding the long term impact of this therapy are warranted. Furthermore, we believe it is important to follow PTNS treatment outcomes longitudinally to determine if it is effective in a progressive disease such as MS. Previous published PTNS studies did not address how efficacy could be affected by the type of MS, disability status or whether the efficacy could be maintained long term in the setting of a progressive disease.

Therefore, the aim of this study is to describe short- and long-term clinical outcomes, including quality of life, bladder, bowel and sexual function, in MS patients who undergo PTNS treatment for refractory LUTS.

## Methods/Design

### Study design and population

The study is registered with clinicaltrials.gov, NCT04063852. This study is designed as a single-centered, prospective, longitudinal cohort study with plans to follow participants for up to 24 months. Adult patients at the University of Michigan outpatient urology clinic presenting with multiple sclerosis and lower urinary tract symptoms (LUTS) including urinary frequency, urgency, incontinence refractory to two prior treatments who have elected to pursue percutaneous PTNS will be invited to participate (Table 2). Refractory symptoms are defined as bothersome LUTS that have not responded to at least two prior therapies including behavior modification, pharmacotherapy, pelvic floor physical therapy or bladder onabotulinum toxin. Full inclusion and exclusion criteria are listed in Table 2.

**Table 2:** Inclusion and Exclusion criteria

**Inclusion Criteria**	
Adults (age greater than or equal to 18 years old)	
Diagnosis of Multiple Sclerosis	
Lower urinary tract symptoms (urinary frequency, urgency and/or incontinence)	
Failed prior first and second line therapy (behavioral and pharmacotherapy)	
Electing for Posterior Tibial Nerve Stimulation therapy for urinary symptoms. Patients performing Intermittent Catheterization are Eligible	
**Exclusion Criteria**	
Age less than 18 years	
Indwelling catheters (urethral or suprapubic)	
currently pregnant or planning pregnancy	
Unable to attend weekly office visits for PTNS	
urodynamic findings of bladder outlet obstruction	
History of:	
bladder reconstruction (augmentation cystoplasty, catheterizable channel)	
cystectomy	
bladder stones	
pacemaker or defibrillator	
malignancy of bladder	
sacral neuromodulation	
intravesical injection of onabotulinum toxin within 9 months	

The PTNS procedure will be performed per the manufacturer’s specifications using a Neuromodulation System (Urgent® PC, Minnetonka, MN), as previously described [15]. Participants will undergo treatment for 30 min, once per week for 12 weeks. After 12 weeks, those with more than 50% improvement in symptoms based on a visual analog scale, will be offered maintenance PTNS sessions for 30 min every 28 days for a total of 24 months.

### Recruitment

Participants will be recruited via a three-pronged approach. First, neurourology clinics at a tertiary care referral center will be screened for eligible patients. Eligible patients will be mailed letters regarding the study. Second, neurology and physical medicine and rehabilitation physicians at the same institution will be notified of the study. Finally, advertisements for the study will be posted on social media networks.

### Screening and enrollment

Patients who meet inclusion criteria will be evaluated at baseline. Demographic and social information, and complete history and physical and neurologic evaluation with the Expanded Disability Status Scale (EDSS) will be evaluated at this time. Information from prior brain and spine MRI studies will be collected, if available. Participants will complete baseline questionnaires outlined below and summarized in Table 3.

**Table 3:** Outcome measurements and time points

	Time Points (month)			
	0*	3**	12	24
**Demographic Intake Form**	x			
**LUTS Measurements:**				
**3 Day Voiding Diary**	x	x	x	x
**AUA-SS**	x	x	x	x
**M-ISI**	x	x	x	x
**NBSS**	x	x	x	x
**Visual Analog Scale**		x		
**Sexual Satisfaction Scale**	x	x	x	x
**Bowel Control Scale**	x	x	x	x
**SF-12**	x	x	x	x
**Expanded Disability Scale**	x		x	x

*Time 0: start of PTNS therapy; **Time 3 months: end of weekly PTNS therapy; *AUA-SS American Urological Association Symptom Score, *M-ISI* Michigan Incontinence Symptom Index, *NBSS* Neurogenic Bladder Symptom Score

### Follow-up and retention

Efforts will be made to follow-up all patients through 24 months after time 0 (initiation of PTNS). This is inclusive of patients who do not have an improvement of symptoms with initial 12 weeks of PTNS and do not pursue maintenance PTNS. In order to promote follow-up and retention, participants will be offered the opportunity to complete questionnaires in person or electronically, via a Research Electronic Data Capture (REDCap) [25]. Furthermore, participants will be compensated at the time of return of a 3-day voiding diary at times 0, 12, and 24 months.

### Collection of study data

Study data will be collected and managed using REDCap hosted at the University of Michigan [25]. REDCap is a secure, web-based application designed to support data capture for research studies [25].

### Study measurements

Eligible persons will be evaluated at baseline (time of first PTNS treatment) and then at months 3, 12 and 24 from baseline (Table 3). Patients will be administered instruments assessing LUTS, HRQOL, Bowel and Sexual symptoms. Data collection instruments are summarized in Table 4.

**Table 4:** Description of data collection instruments

	Name	Description
**Urinary Measures**	3-day Voiding Diary	Patient completed 3 day assessment of fluid intake, output, incontinence episodes and pad changes.
	American Urological Association Symptom Score (AUA-SS)	Validated symptom score for the evaluation of Lower Urinary Tract Symptoms in Benign Prostatic Hyperplasia. Has been used in MS related research in the past.
	Michigan Incontinence Symptom Score (M-ISI)	Validated score developed to discern between type of incontinence and the severity and bother caused by urinary incontinence
	Neurogenic Bladder Symptom Score (NBSS)	Objective and validated assessment of bladder symptoms specifically created for use in patients with neurogenic bladder
**Bowel and Sexual Measures**	Sexual Satisfaction Scale	Validated, 5 item index addressing overall sexual adjustment in patients with MS and was adapted by the MSQLI from the Sexual History Form, scores range from 4 to 24 with higher scores indicating greater problems with sexual satisfaction.
	Bowel Control Scale	validated, 5 item scale evaluating constipation, bowel accidents, bowel urgency and the impact of bowel symptoms of lifestyle on a 25 point scale with higher scores indicating greater bowel problems
**General HRQOL**	SF-12	Validated 12 item quality of life survey for use in patients with chronic conditions.
**MS Impact**	Expanded Disability Scale	Physician Completed assessment of impact of MS

*HRQOL* Health Related Quality of Life. *MS* Multiple Sclerosis

#### Description of urinary symptom assessment tools

For evaluation of LUTS the following instruments will be utilized: 1) 3-day voiding diary, 2) American Urological Association Symptom Score (AUA-SS), 3) Michigan Incontinence Symptom Index (M-ISI), and 4) Neurogenic Bladder Symptom Score. Voiding diaries are recommended in the routine evaluation of patients with neurogenic overactive bladder and the 3 day diary has been shown to be reliable in the evaluation of urge urinary incontinence [26, 27]. The AUA-SS was validated in 1992 and has been widely used in urologic literature including in the assessment of patients with MS. [11, 28, 29] Scores range from 0 to 35 with greater scores indicating more severe symptoms. The M-ISI is a 10 item questionnaire was developed and validated to discern between type of incontinence and the severity and bother caused by incontinence [30]. Greater scores indicate increased symptoms and the minimally important difference for urge urinary incontinence subdomain is 2 points [30]. The M-ISI has been utilized in prior literature in the context of MS. [11] The Neurogenic Bladder Symptom Score is a 22 item survey developed and validated for use in patients with spinal cord injury, MS and spina bifida [31]. The NBSS is a symptom scale which captures data regarding urinary incontinence, storage and voiding symptoms and complications of neurogenic bladder [31]. (Table 4).

#### Description of adjunct assessment tools

Additional information regarding sexual activity and bowel symptoms will be assessed via selected tools which are part of the Multiple Sclerosis Quality of Life Index (MSQLI) [32, 33]. The MSQLI serves as a comprehensive assessment of HRQOL in patients with MS and consists of a Health Status Questionnaire (SF-36) and 9 subscales including bowel and sexual domains [32, 33]. For the purposes of this study the SF-12, Bowel Control Scale and Sexual Satisfaction Scale from the MSQLI are selectively utilized. The SF-12 is a 12-item version of the SF-36, the SF-12, was chosen to assess health status in order to decrease survey fatigue [34]. Literature has shown comparable results between SF-36 and SF-12 in the MS population [34, 35]. The Bowel Control Scale is a 5 item scale evaluating constipation, bowel accidents, bowel urgency and the impact of bowel symptoms of lifestyle on a 25 point scale with higher scores indicating greater bowel problems [33]. The Sexual Satisfaction Scale is a 5-item index addressing overall sexual adjustment in patients with MS and was adapted by the MSQLI from the Sexual History Form, scores range from 4 to 24 with higher scores indicating greater problems with sexual satisfaction [32, 33]. Both the Sexual Satisfaction Scale and the Bowel Control Scale have been widely used and validated as part of the MSQLI in the MS literature [32, 33]. (Table 4).

#### Description of neurologic disability assessment tools

Neurologic impact of multiple sclerosis in participants will be evaluated using the Expanded Disability Status Scale (EDSS) at baseline and annually by a neurologist, resulting in 3 measurements over the 24-month study time frame [36]. Additionally, we will evaluate participants for change in Magnetic Resonance Imaging (MRI) over the 24-month period, including T2 lesion number and location. The EDSS was developed in the 1950s and has long been the standard for assessing MS progression in phase III clinical trials [36]. The EDSS assesses eight neurological exam functional systems; EDSS 0 means normal and 10, death. Aside from EDSS, MRI will provide objective measures of MS disease burden as reflected on T2 lesion load (Table 4).

### Study outcomes

The primary outcome will be change in the median number of urinary frequency and urinary incontinence episodes on a 3-day voiding diary [37]. Secondary outcomes will be change in AUA symptom score (AUA-SS) and bothersome score, Michigan Incontinence Symptom Index (M-ISI), Neurogenic Bladder Symptom Score (NBSS), and from the Multiple Sclerosis Quality of Life Index (MSQLI) the Health Status Questionnaire (SF-12), Sexual Satisfaction Scale (SSS), and Bowel Control Scale (BCS) [28, 30–33].

### Sample size

In this descriptive study, we aim to enroll at least 50 participants. While it is likely that the count of episodes in each of the time frames follows a Poisson distribution, the difference statistic could very well be expected to follow a normal distribution. If not, appropriate transformations would be applied to create an approximately normal distribution. Allowing for up to a 10% dropout rate, this sample size will provide a 95% chance to have a 95% confidence interval for our change statistic that is within +/− 1/3 of a standard deviation. In other words, if we have an underlying standard deviation of 3 for the difference in counts, simulation results indicate that over 95% of the time, the 95% confidence interval for the change statistic would fall within the mean +/− 1. This will provide sufficient precision for our outcomes of interest.

## Statistical analysis

Baseline descriptive demographic data will be presented for the entire cohort. The primary outcome of median number of urinary frequency and incontinence episodes on a 3-day voiding diary at 3, 12 and 24 months will be compared to baseline for each patient using paired analysis. Secondary outcome measures including total AUA-SS, M-ISI, NBSS, SF-12, SSS and BCS scores at 3, 12 and 24 months will be compared to baseline for each patient using paired analysis. Continuous variables will be evaluated using paired t-test and categorical data will be analyzed using McNemar’s test. Paired data will be evaluated using a 2-sided paired t-test. Median values will be analyzed using a Wilcoxon signed rank test. Statistical significance will be considered with *p* < 0.05.

In order to assess for predictors of improvement of symptoms response to PTNS, planned subgroup analysis will be performed stratifying patients based on gender, EDSS score, and burden of disease on MRI. Furthermore, an interim analysis at evaluating outcomes at 3 and 12 months after PTNS is planned.

## Discussion

The current literature regarding outcomes of PTNS in patients with MS who suffer from refractory lower urinary tract symptoms is favorable and is summarized in Table 1 [16–22, 38]. In 2009 Kabay published 3-month clinical outcomes in 19 patients showing significant decrease in mean overactive bladder questionnaire (OAB-V8) scores (15.7 vs 7.6, *p* < 0.05) [18]. Additionally they found a 40% complete response for urinary incontinence, and 58% complete response for urinary frequency on bladder diaries [18]. In 2011, Gobbi reported 3 month results in 18 patients from a multicenter, open label trial which found that there was statistically significant improvement in frequency (9 vs 6 voids, *p* = 0.04) and nocturia (3 vs 1 episodes, *p* = 0.002), additionally there was a 70% treatment satisfaction and significant improvement in the patient perception of bladder condition (PPBC) and patient perception of intensity of urgency scales (PPIUS) as well as the King’s Health Questionnaire (KHQ) [19]. The same group published long term outcomes (mean 24 months, range 15–41) which included variable maintenance PTNS every 2–4 weeks, in 83 patients [20]. They reported 89% had greater than 50% improvement on the PPBC at 12 months and 82% had treatment satisfaction and improvement in PPBC at 24 months [20]. Of the 74 patients undergoing maintenance, the mean monthly treatment frequency was 1.8 sessions [20]. Most recently, Canbaz Kabay published 12-month outcomes in 29 patients, with 21 patients undergoing maintenance PTNS at variable intervals between 2 and 4 weeks [22]. Similar to prior studies, they found significant improvements in frequency, nocturia, urgency, and incontinence at 3, 6, 9 and 12 months of follow-up [22]. Furthermore, they found that there was significant improvement on International Consultation on Incontinence Questionnaire-Short Form (ICIQ-SF), OAB-V8 and OAB-q at 12 months [22].

Prior literature suggests that PTNS will improve LUTS in patients with MS, and that results may be sustained up to 24 months with maintenance therapy. However, the literature is limited in several ways. First, only two prior studies report outcomes beyond 3 months, in a total of 95 patients [20, 22]. Next, the published maintenance schedules for PTNS in MS have varied between every 2–4 weeks, which makes it difficult to assess whether monthly maintenance PTNS is adequate for sustained efficacy [20, 22]. Furthermore, all prior studies have excluded patients with EDSS greater than 7 [16–22, 38]. Since patients with greater EDSS scores have increased disability, the impact of progressive MS patients who require assistive devices or wheelchairs for mobility is unknown. Finally, prior studies did not assess how early MS would respond to treatment. In light of ours and others’ studies showing RRMS are associated with more bothersome urinary symptoms, it will interesting to see how the treatment will affect early MS with EDSS less than 2 and if disease progression impacts PTNS efficacy [7, 23].

Another significant knowledge gap is that health related quality of life (HRQOL) has been reported in only one study and no prior studies have reported on potential improvements in bowel and sexual function with PTNS [19]. Evaluating the impact on HRQOL provides data to guide clinicians and patients on whether the investment in PTNS is worthwhile for overall health. Assessment of bowel and sexual function outcomes may expand the applications for PTNS in this population. Finally, none of the previous PTNS studies have incorporated MRI measures. As another novel exploratory measure, our protocol will also incorporate retrospective MRI data of the participants, obtained for routine neurologic surveillance, before and after PTNS in order to evaluate whether brain and spinal cord lesion location, T2 lesion number and volume changes, and disease burden impact track with PTNS treatments.

## Conclusion

This research protocol aims to expand on the existing literature regarding outcomes of PTNS in MS. Specifically, it will provide 24-month follow-up data on bladder, bowel, sexual and HRQOL outcomes. Further, it will be investigational data correlating EDSS and MRI results to PTNS, to evaluate whether lesion location or burden of disease is predictive of response to PTNS.

## Data Availability

Investigators will publish trial results. The datasets generated and/or analysed during the current study are not publicly available because they are identifiable but are available from the corresponding author on reasonable request.

## Abbreviations

AUA-SS: American Urological Association Symptom Score
BCS: Bowel Control Scale
CIS: Clinically isolated syndrome
EDSS: Expanded Disability Status Scale
HRQOL: Health related quality of life
KHQ: King’s Health Questionnaire
LUTS: Lower urinary tract symptoms
M-ISI: Michigan Incontinence Symptom Index
MRI: Magnetic resonance imaging
MS: Multiple Sclerosis
MSQLI: Multiple Sclerosis Quality of Life Index
NBSS: Neurogenic Bladder Symptom Score
OAB: Overactive bladder
PPBC: Patient perception of bladder condition
PPIUS: Patient perception of intensity of urgency scales
PPMS: Primary progressive multiple sclerosis
PTNS: Posterior tibial nerve stimulation
RRMS: Relapsing remitting multiple sclerosis
SF-12: Health Status Questionnaire
SPMS: Secondary progressive multiple sclerosis
SSS: Sexual Satisfaction Scale
